# Intestinal Diseases in Children

**Published:** 1901-10

**Authors:** Geo. Howard Thompson

**Affiliations:** Professor of Materia Medica and Experimental Medicine, St. Louis College of Physicians and Surgeons


### Intestinal Diseases in Children.

BY GEO. HOWARD THOMPSON, M. D.,
Professor of Materia Medica and Experimental Medicine, St. Louis College of Physicians and Surgeons.

Dentition, in my opinion, is only incidental to the gastro-intestinal disturbances known under the general name of cholera infantum. True it is that children are highly susceptible to reflex disturbances prompted by irritation in the gums at the teething period. Any constant irritation, however, may produce the same reflex disturbances. Cholera infantum coincidental with teething is apt to be regarded as a result of this physiological process by the thoughtless observer. We must differentiate between a coincidence and a cause. We must differentiate between a coincidence and a contributing cause. Cholera infantum may take place at any season of the year; so may the process of teething. These two conditions frequently take place simultaneously. Cholera infantum, however, is far more likely to take place in the summer—in the central and southern sections in August and September—than in any other season of the year. Indiscretions in diet are the exciting causes. When teething is coincidental we can more readily understand the influence of indiscretion than at a later period. The mere fact of dentition can be responsible only for certain reflex nervous disturbances; but the cause of dentition is the necessity for a change in the child's diet. The child must be supplied with food different from mother's milk, otherwise, why the advent of teeth? This necessity is recognized intuitively by the mother, who, not knowing how to meet the indication physiologically, is only too apt to kill the child with mistaken kindness. The child gets bread to bite which the child's gastro-intestinal apparatus is not ready to dispose of properly. It ferments and sets up a gastro-intestinal irritation, nausea, vomiting and purging results, and finally the doctor is called.

This summer, I have had a large number of cases of cholera infantum, subacute gastro-intestinal indigestion and marasmus. Probably my best results were derived from the following line of treatment:

Baby was given podophyllin, one-fortieth of a grain, or calomel, one-tenth of a grain, until the passages become distinctly bilious. Then an intestinal antiseptic sedative and astringent consisting of:

℞ Bismuth subcarbonatis ..................... dr. i
Glyco-Thymoline (Kress) ............... fl. oz. ss
Misturae cretae................. q. s. ad. fl. oz. iij

M. Sig.—Shake the bottle and take a teaspoonful every three hours.

It is always a good idea to give the baby an enema of a pint of warm water, in which is an ounce of Glyco-Thymoline, at the commencement of treatment, in order that the colon may be relieved as rapidly as possible of its fermenting and decomposing contents, and the lining meet the antiseptic and healing application as soon as possible. Daily enemata of the above solution are frequently advisable.

This summer, I have had fewer unfortunate terminations in my cases of cholera infantum than in previous years, and I attribute my good fortune largely to the fact that, at the instigation of Dr. A. E. Chatfield, of Cleveland, Ohio, I have added this non-irritating antiseptic Glyco-Thymoline to my internal medication in inflammatory diseases of the gastro-intestinal tract.

It has proven to have these advantages: It is antiseptic; it is non-toxic in the dose given; it is non-irritant; it is healing. It is about the only antiseptic that can be safely given to children.

